# Multiple imputation using linked proxy outcome data resulted in important bias reduction and efficiency gains: a simulation study

**DOI:** 10.1186/s12982-017-0068-0

**Published:** 2017-12-19

**Authors:** R. P. Cornish, J. Macleod, J. R. Carpenter, K. Tilling

**Affiliations:** 1 Population Health Sciences, Bristol Medical School, University of Bristol, Oakfield House, Oakfield Grove, Bristol, UK; 2 Integrative Epidemiology Unit, University of Bristol, Bristol, UK; 3 Department of Medical Statistics, Faculty of Epidemiology and Population Health, London School of Hygiene and Tropical Medicine, London, UK; 4 MRC Clinical Trials Unit, Institute of Clinical Trials and Methodology, School of Life and Medical Sciences, University College London, London, UK

**Keywords:** Missing data, Multiple imputation, Bias, Simulation study, ALSPAC, Data linkage, Breastfeeding, IQ

## Abstract

**Background:**

When an outcome variable is missing not at random (MNAR: probability of missingness depends on outcome values), estimates of the effect of an exposure on this outcome are often biased. We investigated the extent of this bias and examined whether the bias can be reduced through incorporating proxy outcomes obtained through linkage to administrative data as auxiliary variables in multiple imputation (MI).

**Methods:**

Using data from the Avon Longitudinal Study of Parents and Children (ALSPAC) we estimated the association between breastfeeding and IQ (continuous outcome), incorporating linked attainment data (proxies for IQ) as auxiliary variables in MI models. Simulation studies explored the impact of varying the proportion of missing data (from 20 to 80%), the correlation between the outcome and its proxy (0.1–0.9), the strength of the missing data mechanism, and having a proxy variable that was incomplete.

**Results:**

Incorporating a linked proxy for the missing outcome as an auxiliary variable reduced bias and increased efficiency in all scenarios, even when 80% of the outcome was missing. Using an incomplete proxy was similarly beneficial. High correlations (> 0.5) between the outcome and its proxy substantially reduced the missing information. Consistent with this, ALSPAC analysis showed inclusion of a proxy reduced bias and improved efficiency. Gains with additional proxies were modest.

**Conclusions:**

In longitudinal studies with loss to follow-up, incorporating proxies for this study outcome obtained via linkage to external sources of data as auxiliary variables in MI models can give practically important bias reduction and efficiency gains when the study outcome is MNAR.

**Electronic supplementary material:**

The online version of this article (10.1186/s12982-017-0068-0) contains supplementary material, which is available to authorized users.

## Background

In a longitudinal study, where attrition is generally inevitable, knowledge about the most likely mechanism for the missing data is important as this helps the researcher determine an appropriate strategy for the statistical analysis in order to minimise bias and maximise efficiency. There are several approaches to analysing datasets containing missing information. The most widely used are a complete records analysis, in which only subjects with fully observed data are included, and multiple imputation (MI). In MI, a number of complete datasets are created in which missing values are replaced by imputed values using models fitted to the observed data. Standard statistical models are then used to analyse each dataset, and the estimates obtained from these are then combined appropriately [1]. In a longitudinal study with attrition, the data for any given analysis are likely to be either missing at random (MAR) or missing not at random (MNAR). Data are MAR if, after taking account of observed variables, the probability that they are missing does not depend on the (unknown) missing values, and MNAR if this probability does depends on the missing values even after taking account of the observed data. If data are MAR, then a complete records analysis will produce an unbiased estimate of the exposure-outcome relationship only if missingness is unrelated to the outcome variable and all observed variables associated with missingness are included in the analysis model. MI will produce an unbiased estimate as long as all observed variables associated with missingness are included in the imputation model [1]. Conversely, if the data are MNAR, then a standard implementation of MI will give a biased estimate of the exposure-outcome relationship whereas a complete records analysis will generally produce an unbiased (but inefficient) estimate as long as missingness is unrelated to the outcome (and for additional situations if the outcome is binary and logistic regression is used for the analysis) [1, 2].

Generally, the missing data mechanism cannot be determined from the study data alone. In particular, it is not possible to distinguish between data that are MAR and data that are MNAR. However, if a proxy for the missing variable is available through linkage to an administrative data source whose coverage amongst eligible individuals is greater than that of the study data, then a set of plausible missingness mechanisms can be identified. These proxies can also be used as auxiliary variables in multiple imputation (MI) and other models used to take account of missing data. (Auxiliary variables are variables that are associated with missingness as well as with the variable(s) with missing values, but are not included in the substantive model.) In recently published work [3] we used data on educational attainment at age 16 years—obtained via linkage to the National Pupil Database (NPD), a longitudinal database containing attainment and other data for children attending schools in England—to investigate the missingness mechanism for IQ measured at age 15 in ALSPAC. We also used attainment data as additional predictors (auxiliary variables) in MI models and in the calculation of inverse probability weights. This allowed us to study the association between duration of breastfeeding and IQ at 15 years. In the current paper we extend this example using the ALSPAC data by including earlier educational attainment—measured at Key Stages 2 and 3—as additional auxiliary variables in the multiple imputation models and present these results. We then describe a simulation study, based on this example, to examine the extent of the bias in the exposure-outcome relationship—where the outcome is a continuous variable—and to further explore the conditions under which linking to a proxy outcome variable (and using it as an auxiliary variable in MI models) reduces this bias. We vary the degree of correlation between the original outcome and its proxy, the proportion of missing data, and the extent to which the outcome is MNAR.

## Methods

ALSPAC provided the motivating example for this simulation study. ALSPAC is a birth cohort which recruited c14,500 pregnant women living in and around the city of Bristol, in the south west of England, in the early 90 s. Detailed data were collected during pregnancy and the offspring have been followed up since birth through questionnaires, study clinics and linkage to health and administrative datasets. Further details are given in the cohort profile paper [4]. ALSPAC has a searchable data dictionary describing all available data [5].

### Analysis of ALSPAC data

#### What we did previously

In our previous analysis [3] we included the following variables from ALSPAC: the outcome was IQ measured at 15 years using the Wechsler Abbreviated Scale of Intelligence (WASI) [6]; the exposure was duration of breastfeeding, derived from questionnaires administered at 4 weeks, 6 and 15 months. Other variables (confounders) adjusted for were baseline covariates, including the child’s sex and ethnicity, smoking during the first trimester of pregnancy, maternal age and parity (number of previous births), mother’s and father’s educational level, family occupational social class, housing tenure (whether the family home was owned/mortgaged, privately rented, or rented from the local council or a housing association), and family adversity index, a composite measure of social adversity. Finally, two Key Stage 4 (KS4) attainment variables from the NPD were included as auxiliary variables—both in MI and for calculating inverse probability weights: the number of A*–C grades obtained, dichotomised as < 5 or 5 or more, and the capped KS4 point score, the total score of an individual’s top eight GCSE or equivalent qualifications ranked in terms of points. Key Stage 4 refers to the two school years attended between 14 and 16 years. In this period pupils typically take GCSE (General Certificate of Secondary Education) or equivalent vocational courses in a number of subjects and then receive a grade in each. Each grade is equivalent to a specified number of points, higher scores indicating higher attainment.

#### Additional analyses in the current study

In the current study we extend our previous analysis by including two additional linked attainment variables—a Key Stage 2 (KS2) attainment score and a Key Stage 3 (KS3) attainment score. In both cases, these variables were derived from National Curriculum test results in English, maths and science. KS2 tests are taken at the end of primary school, the school year in which children have their 11th birthday; KS3 tests are taken at the end of year 9, when children are aged 13 or 14 years. In English, pupils have a reading test, which is scored out of 50 and a writing assessment (out of 50). In maths there are two papers, both scored out of 40 in KS2 and out of 60 in KS3, and a mental arithmetic test (out of 20 in KS2 and 30 in KS3); finally, in science there are two papers, both scored out of 40 in KS2 and out of 90 in KS3 (science tests were discontinued at Key Stage 2 in 2010 [7], but were sat by ALSPAC children). The KS2 and KS3 attainment scores were obtained by adding together the English, maths and science scores, thus giving a maximum possible attainment score of 280 in KS2 and 430 in KS3.

As in our previous analysis, fractional polynomials [8] were used to obtain the best fitting models for predicting IQ from the KS2 and KS3 attainment scores. For simplicity we chose the best fitting one power model for each; this gave a quadratic model predicting IQ from both the KS2 attainment score and the KS3 attainment score. As reported previously [3], the best fitting model predicted IQ from the KS4 attainment score cubed. Multiple imputation using chained equations was used to model the relationship between breastfeeding and IQ. These models have been described in detail previously [3]. In addition to the variables included in our initial analysis, IQ was imputed from the KS2 attainment score squared and the KS3 attainment score squared (in addition to the cubed KS4 attainment score); similarly the individual attainment scores at KS2 and KS3 were imputed from the square root of IQ (in addition, and as previously, the KS4 attainment score was imputed from the cube root of IQ). Note that none of the attainment scores were in the substantive model, but were only included as auxiliary variables when imputing missing values. Stata’s *mi impute chained* command was used to carried out the imputations; 100 datasets were imputed.

### Simulated datasets

As detailed below, our simulations were based on the ALSPAC data. The variables we simulated were analogous to IQ (the outcome variable), duration of breastfeeding (the exposure), offspring sex and mother’s education. We simulated a single proxy (attainment) variable; for simplicity we will think of this as analogous to the Key Stage 4 attainment score, as this was the main attainment variable included in our original paper. We will refer to this as the linked attainment score in the remainder of this paper.

We first simulated complete datasets. Missing data were then simulated in a separate process. We simulated datasets of 10,000 observations—which approximately matches the numbers in ALSPAC with complete baseline covariates—in which we had four variables with distributions chosen to be roughly representative of those seen in ALSPAC. Sex and mother’s education were the two covariates. Sex was simulated as a binary variable with probability 0.5 of being male/female and mother’s education as a categorical variable with probabilities 0.5, 0.25 and 0.25 of being in categories O level or lower, A level, and university degree or higher (respectively). (In England and Wales, A levels are exams usually taken at age 18; O levels used to be exams taken at age 16 but these were replaced by GCSEs in 1988). The exposure variable, duration of breastfeeding, was created as a categorical variable, with categories designed to represent: never/less than one month, 1– < 3 months, 3– < 6 months, and 6 + months. This variable was simulated as being dependent on mother’s education such that duration of breastfeeding increased with higher maternal education. The marginal probabilities for the four breastfeeding categories were: (0.5,0.15,0.15,0.2), (0.3,0.1,0.2,0.4) and (0.15,0.1,0.15,0.6) for O level/lower, A level, and degree/higher, respectively. The outcome, IQ at age 15 years, was simulated as a standard normal variable (i.e. a normal variable with mean equal to 0 and variance 1), dependent on sex, mother’s education and duration of breastfeeding such that:

1 $$\begin{aligned} {\text{IQ}} & = \beta_{0} + \beta_{1} \left( {sex} \right) + \beta_{2} \left( {mumed_{1} } \right) + \beta_{3} \left( {mumed_{2} } \right) \\ & \quad + \beta_{4} \left( {BF_{1} } \right) + \beta_{5} \left( {BF_{2} } \right) + \beta_{6} \left( {BF_{3} } \right) + \varepsilon \\ \end{aligned}$$

where *sex* is the indicator variable for sex, *mumed*_1_ and *mumed*_2_ for the mother having A levels and a degree level qualification or higher, respectively, *BF*_1_, *BF*_2_ and *BF*_3_ are the indicator variables for being breastfed for 1 to < 3, 3 to < 6 and 6 months or longer, and $\varepsilon$ is the random error, following a normal distribution with mean 0 and variance $\sigma^{2}$, with the latter calculated to give IQ a variance of 1. The coefficients of this regression model were fixed to be as follows: β_0_ = − 0.4, β_1_ = −0.1, β_2_ = 0.4, β_3_ = 0.8, β_5_ = 0.1, β_6_ = 0.2, β_7_ = 0.3, representing relationships similar to those seen in ALSPAC. This was also the analysis model. The linked attainment score was also simulated as a standard normal variable (mean 0, variance 1). For simplicity, this was made dependent (with a linear relationship) only on IQ:

2 $$KS4 = \rho \left( {IQ} \right) + \tau$$

with *KS4* representing the linked (Key Stage 4) attainment score and $\tau$ the (normal) random error with mean 0 and variance $\varphi^{2}$, again calculated to give the attainment score a variance of 1. Both IQ and the linked attainment score were simulated to have mean 0 and variance 1 in order that our results would not be influenced by the scale of these measures—and thus would be generalizable to other continuous measures. In a sensitivity analysis we also made the attainment score dependent on sex and mother’s education in addition to IQ.

### Generating the missing data

Because the focus of this study was the utility of proxy data for missing outcomes, we only created missing data in the outcome variable, IQ. Firstly, we simulated IQ as being MAR. We then made IQ MNAR. We did not consider MCAR (missing completely at random, probability of being missing does not depend on either observed or unobserved variables) since this would be very unlikely to occur in the context of dropout in epidemiological studies. The probabilities were generated using a binomial regression model, again with coefficients similar to those seen in ALSPAC:

3 $$\begin{aligned} {\text{Pr(IQ}}\;{\text{observed)}} & = \alpha + \gamma_{1} \left( {sex} \right) + \gamma_{2} \left( {mumed_{1} } \right) + \gamma_{3} \left( {mumed_{2} } \right) \\ & \quad + \gamma_{4} \left( {BF_{1} } \right) + \gamma_{5} \left( {BF_{2} } \right) + \gamma_{6} \left( {BF_{3} } \right) + \gamma_{7} \left( {IQ} \right) \\ & \quad + \gamma_{8} \left( {BF_{1} \times IQ} \right) + \gamma_{9} \left( {BF_{2 } \times IQ} \right) + \gamma_{10} \left( {BF_{3} \times IQ} \right) \\ \end{aligned}$$

Some of the regression coefficients in this model were fixed throughout the simulation study: γ_1_ = 0.04 (female compared to male), γ_2_ = 0.075 (mother’s education = A level compared to mother’s education = O level or lower), γ_3_ = 0.10 (mother’s education degree or higher compared to O level or lower), γ_4_ = 0.08 (breastfed for 1 to < 3 months compared to never/less than one month) γ_5_ = 0.12 (breastfed for 3 to < 6 months compared to never/< one month) and γ_6_ = 0.14 (breastfed for 6 + months compared to never/< one month). The remaining coefficients were varied. The values of α were first calculated and then adjusted, where necessary, using a trial and improvement method in order to produce particular percentages of missing data; details are given in the Scenarios section. These adjustments using trial and improvement were necessary for scenarios in which Eq. (3) produced negative predicted probabilities.

Because a binomial model was used to predict the probability of IQ being missing, this sometimes led to negative predictions; this was particularly the case when simulating datasets with 80% missing data. When the probability was predicted as negative, IQ was automatically set to missing; if not, for each observation a Bernouilli random variable with p = Pr(IQ observed) was drawn to determine whether each IQ was missing.

Finally, we simulated the linked attainment score to be either complete or MNAR, with missingness only dependent on itself; again, the probabilities were generated using a binomial regression model:

4 $${ \Pr }\left( {{\text{KS4 }}\;{\text{observed}}} \right) = \pi + \delta \left( {KS4} \right)$$

The value of the intercept, π, was set at 0.8 in order to produce 20% missing linked attainment data. Values of δ were varied. As for IQ, a Bernouilli random variable with p = Pr(KS4 observed) was drawn and used to determine whether each record was set to missing.

### Scenarios

Key factors influencing the extent of bias are the amount of missing data and the degree to which the outcome is MNAR; the strength of association between the outcome and its proxy will largely determine the degree to which this bias can be reduced. Thus, these constituted the three primary factors varied in the simulations. These were varied as detailed below.

- Factor 1: The percentage of missing outcome (IQ) data: 20, 40, 60, 80%.
- Factor 2: How good a proxy the linked variable was: correlation between the outcome variable (IQ) and its linked proxy (attainment score) = 0.1, 0.3, 0.5, 0.7, 0.9.
- Factor 3: Whether the outcome (IQ) was MAR or MNAR and, if the latter, the extent of this: increase in probability of observing IQ for a one SD increase in IQ = 0 (MAR), 0.05, 0.1, and 0.2 [$\gamma_{7}$ from Eq. (3)].

In addition we hypothesised that if the association between IQ and the probability if it being missing varied according to duration of breastfeeding this would substantially increase bias. Thus, our first secondary factor was:

- Factor 4: Whether or not the association between the outcome (IQ) and the probability of it being observed differed according to the exposure (breastfeeding): $\gamma_{8}$ from Eq. (3) = 0 or − 0.025. For simplicity, we made the strength of association between IQ and the probability of it being missing change linearly with increasing breastfeeding, such that:

$$\gamma_{9} = 2\gamma_{8 }\,{\text{and }}\,\gamma_{10} { = 3}\gamma_{8} \; \left( {{\text{from}}\;{\text{equation}}\;\left( 3 \right)} \right)$$

Finally, in our main sets of scenarios there was no missingness in the linked variable.

However, we also wanted to consider some scenarios in which the linked proxy was not available for all individuals and therefore introduced missingness in this variable. This formed our other secondary factor:

- Factor 5: Whether or not there was missingness in the linked attainment score and varying the direction of missingness: difference in probability of Pr(KS4 observed) for a one SD increase in attainment score (KS4) = − 0.10, + 0.10.

The scenarios are summarised in Table 1. We did not consider every possible combination of these factors. The main set of scenarios involved only the three primary factors listed above. However, at each of the four levels of missing data the MAR condition was only simulated in one scenario, with a correlation of 0.7 between IQ and the linked attainment score. This was because our focus in this study was primarily on reducing bias with an outcome variable that is MNAR. We included MAR in the simulations simply to show that the complete case analysis and MI would both be unbiased and that MI would simply increase efficiency in this situation. Additional scenarios involved our two secondary factors but these were only introduced for a limited set of scenarios (Table 1). Altogether, there were 100 scenarios. For each scenario, 1000 datasets were simulated.

**Table 1:** Scenarios investigated in the simulations (each investigated with 20, 40, 60 and 80% missing outcome data)

			Factor 3: Change in Pr(IQ observed) for one SD increase in IQ	Factor 2: Correlation between IQ and linked attainment score (KS4)				
				0.1	0.3	0.5	0.7	0.9
Main set of scenarios (each at 20, 40, 60, 80% missing IQ (factor 1)): 64 scenarios								
			0				✓	
			0.05	✓	✓	✓	✓	✓
			0.1	✓	✓	✓	✓	✓
			0.2	✓	✓	✓	✓	✓
Secondary sets of scenarios (each at 20, 40, 60, 80% missing IQ (factor 1)): 36 scenarios								
Missing linked data	Factor 5: Change in Pr(KS4 observed) for one SD increase in KS4	Factor 4: Association between IQ and Pr(IQ observed) dependent on breastfeeding?						
No	–	Yes	0.1^a^	✓	✓	✓	✓	✓
Yes, 20%	− 0.1	No	0.1			✓	✓	
	+ 0.1		0.1			✓	✓	

^a^In the baseline breastfeeding group; reduction in this coefficient of 0.025 for each consecutive breastfeeding group

### Statistical analysis

We estimated the coefficients for breastfeeding (β_4_, β_5_ and β_6_) using the multiple linear regression model given by Eq. (1). These were determined using:

- A complete records analysis
- Multiple imputation. For each simulated dataset, 100 imputed datasets were created. The imputation models included all the variables specified above—i.e. all the variables included in the analysis model plus the linked attainment score.

The estimates obtained from these analyses were compared to the true parameters 0.1, 0.2 and 0.3. For each parameter, β_i_, the bias was estimated as $\bar{b}_{i} - \beta_{i}$, where $\bar{b}_{l}$ is the estimated regression coefficient for parameter β_i_ averaged over the 1000 simulated datasets. This was converted to percentage bias. We also calculated the mean squared error (MSE) and the empirical standard error, the standard deviation of the point estimates for each parameter. In addition, for the analyses using multiple imputation, we calculated the fraction of missing information (FMI) for each coefficient and the percent increase in precision compared to the complete records analysis; the latter is given by the variance of the point estimates for the parameter of interest obtained using a complete records analysis divided by the variance obtained using multiple imputation.

The simulations and all data analysis were carried out in Stata 13.0.

## Results

### Analysis of ALSPAC data

There were 13,975 subjects included in our original study of whom 11,414 had complete KS4 attainment data. In our previous paper we gave information on numbers with missing IQ, breastfeeding and covariates according to availability of KS4 attainment data [3]. Table 2 gives the numbers, among those with and without KS4 attainment data, according to availability of KS2 and KS3 attainment data. As previously, the most common missing data pattern was missing outcome only (n = 4405).

**Table 2:** Availability of additional linked attainment data according to presence/absence of KS4 data among the 13,975 subjects from ALSPAC included in this analysis

	KS2 data available	
	Yes	No
KS4 data = yes (11,414)		
KS3 data available		
Yes	9152	339
No	1511	412
KS4 data = No		
KS3 data available		
Yes	79	10
No	473	1999

To inform the imputations, we used fractional polynomials to find the best fitting one power models predicting IQ from the attainment scores; the best fitting one-power models predicted IQ from KS2 and KS3 attainment scores squared. In our original analysis [3] the two KS4 variables explained 39% of the variability in IQ (adjusted R-squared = 0.39); the addition of the KS2 and KS3 variables increased this to 44%. The three right-hand columns of Table 3 give the results obtained when adding the KS2 and KS3 attainment scores as additional auxiliary variables in the multiple imputation models. The estimates obtained from the complete records analysis and the multiple imputation models including KS4 attainment only were presented in our previous paper [3] but are included here for comparison, with the addition of the percent increase in precision and the FMI. The addition of KS2 and KS3 attainment scores resulted in small additional gains in precision, FMI and increases in the estimates of the effect of breastfeeding on IQ.

**Table 3:** Relationship between duration of breastfeeding and IQ at 15

	Duration of breast-feeding	Analysis approach						
		Complete records analysis (n = 4152)	Multiple imputation, using only KS4 attainment^b^ (n = 13,975)			Multiple imputation, using KS2, KS3 and KS4 attainment^c^ (n = 13,975)		
		Difference in mean IQ (95% CI)	Difference in mean IQ (95% CI)	Gain in precision^d^ (%)	FMI^e^ (%)	Difference in mean IQ (95% CI)	Gain in precision^d^ (%)	FMI^e^ (%)
Unadjusted results	Never/< 1 month	–	–	–	–	–	–	–
	1 to < 3 months	1.9 (0.6, 3.2)	3.2 (2.2, 4.3)	47	60	3.4 (2.4, 4.4)	65	55
	3 to < 6 months	5.1 (4.0, 6.3)	6.6 (5.6, 7.6)	36	58	6.8 (5.9, 7.8)	43	56
	6 months +	7.5 (6.6, 8.5)	9.3 (8.5, 10.1)	38	59	9.6 (8.8, 10.3)	58	53
Adjusted^a^ results	Never/< 1 month	–	–	–	–	–	–	–
	1 to < 3 months	0.8 (− 0.4, 2.0)	1.3 (0.3, 2.4)	36	63	1.5 (0.5, 2.4)	54	58
	3 to < 6 months	2.6 (1.5, 3.7)	3.2 (2.2, 4.2)	26	61	3.4 (2.4, 4.3)	27	62
	6 months +	(2.5, 4.4)	4.2 (3.4, 5.0)	36	58	4.4 (3.6, 5.2)	39	57

^a^Adjusted for sex, maternal and paternal education, occupational social class, parity, maternal age, ethnicity, family adversity index, smoking in pregnancy and housing tenure during pregnancy

^b^IQ predicted from KS4 points cubed (best fitting fractional polynomial of degree 1), plus all other factors. Imputation model for IQ also included an interaction between KS4 points cubed and mother’s education

^c^IQ additionally predicted from KS2 points squared and KS3 points squared

^d^Relative to complete records analysis

^e^FMI: Fraction of missing information

### Simulation study results

As expected, when the data were simulated as missing at random there was no bias in either complete records or the multiply imputed analyses. Multiple imputation—with the linked attainment score as an auxiliary variable—increased precision in all cases where the data were MAR, although the increases were relatively small when the percentage of missing information was low (Table 4).

**Table 4:** Results when IQ simulated as MAR (factor 3 in scenarios)

Scenario (factors 1 and 2)	Complete records			MI including linked attainment score (KS4)				
	Estimate (empirical SE)	% bias	MSE	Estimate (empirical SE)	% bias	MSE	Gain in precision^a^ (%)	FMI (%)
IQ 20% missing Correlation_(IQ:KS4)_ = 0.7	0.1005 (0.033)	0.5	0.001	0.1004 (0.031)	0.3	0.001	10	15
	0.1990 (0.030)	− 0.5	0.0009	0.1993 (0.029)	− 0.3	0.0008	11	13
	0.3006 (0.025)	0.2	0.0006	0.3002 (0.024)	0.1	0.0006	7	13
IQ 40% missing	0.0994 (0.038)	− 0.6	0.001	0.1004 (0.034)	0.3	0.001	22	30
Correlation_(IQ: KS4)_ = 0.7	0.1988 (0.035)	− 0.6	0.001	0.1996 (0.033)	− 0.2	0.001	17	28
	0.3004 (0.030)	0.1	0.0009	0.3004 (0.027)	0.1	0.0008	17	29
IQ 60% missing	0.1005 (0.049)	0.5	0.002	0.1009 (0.042)	1.1	0.002	34	50
Correlation_(IQ: KS4)_ = 0.7	0.1975 (0.042)	− 1.3	0.002	0.1980 (0.037)	− 0.8	0.001	33	47
	0.2988 (0.037)	− 0.4	0.001	0.3002 (0.032)	0.1	0.001	33	48
IQ 80% missing	0.1040 (0.073)	4.0	0.005	0.1050 (0.061)	4.8	0.004	41	83
Correlation_(IQ: KS4)_ = 0.7	0.2009 (0.062)	0.4	0.004	0.2000 (0.052)	0	0.003	40	81
	0.3011 (0.056)	0.4	0.003	0.3022 (0.046)	0.8	0.002	47	81

*MSE* mean squared error, *FMI* fraction of missing information

^a^Relative to complete records analysis

When the outcome variable was simulated as MNAR, both the complete records analysis and the multiple imputation produced biased results; the bias increased as the percentage of missing data increased (Table 5). The results for 80% missing are likely to have been affected by having negative predictions for the probability of IQ being observed—referred to in the methods section. In this scenario, an average of 1156 individuals had a predicted value of Pr(IQ observed), as given by Eq. (3), that was negative and their IQ was thus set to missing. The IQs among these individuals whose predicted probability of being missing was negative were generally low—the mean of the mean IQs in the 1000 datasets was − 1.42 (z-score) and all were below − 0.50—suggesting that, in the scenarios with 80% missing data, the MNAR mechanism was more extreme. The results from the multiple imputation models were less biased and more precise than the complete records analysis in all scenarios where the outcome was MNAR except when the correlation between IQ and the linked attainment score was low (0.1); a correlation of 0.3 gave only small gains in terms of bias and precision. When the correlation was 0.1 the results from multiple imputation were very similar to those from the complete case analysis.

**Table 5:** Results for IQ MNAR: difference in Pr(IQ observed) = 0.10 for 1 SD increase in IQ (factor 3)

Scenario (factors 1 and 2)	Complete records			MI including linked attainment score (KS4)				
	Estimate (empirical SE)	% bias	MSE	Estimate (empirical SE)	% bias	MSE	Gain in precision (%)	FMI (%)
IQ 20% missing	0.08 (0.034)	− 17	0.001	0.08 (0.034)	− 16	0.001	− 0.1	24
Correlation_(IQ:KS4)_ = 0.1	0.17 (0.030)	− 14	0.002	0.17 (0.030)	− 14	0.002	1	21
	0.26 (0.025)	− 12	0.002	0.26 (0.025)	− 12	0.002	0.2	21
IQ 20% missing	As above			0.08 (0.034)	− 15	0.001	2	23
Correlation_(IQ:KS4)_ = 0.3				0.18 (0.030)	− 13	0.001	4	20
				0.27 (0.025)	− 11	0.002	1	20
IQ 20% missing	As above			0.09 (0.033)	− 13	0.001	6	20
Correlation_(IQ:KS4)_ = 0.5				0.18 (0.029)	− 11	0.001	8	18
				0.27 (0.025)	− 9	0.001	4	18
IQ 20% missing	As above			0.09 (0.032)	− 9	0.001	14	16
Correlation_(IQ:KS4)_ = 0.7				0.19 (0.028)	− 7	0.001	14	13
				0.28 (0.024)	− 6	0.001	9	13
IQ 20% missing	As above			0.10 (0.031)	− 4	0.0009	27	7
Correlation_(IQ:KS4)_ = 0.9				0.19 (0.027)	− 3	0.0008	21	6
				0.29 (0.023)	− 2	0.0006	18	6
IQ 40% missing	0.07 (0.041)	− 29	0.003	0.07 (0.041)	− 28	0.002	− 0.2	45
Correlation_(IQ: KS4)_ = 0.1	0.16 (0.035)	− 20	0.003	0.16 (0.035)	− 20	0.003	− 0.5	41
	0.26 (0.029)	− 15	0.003	0.26 (0.029)	− 14	0.003	0.5	42
IQ 40% missing	As above			0.07 (0.040)	− 26	0.002	4	43
Correlation_(IQ: KS4)_ = 0.3				0.16 (0.035)	− 19	0.003	2	40
				0.26 (0.029)	− 14	0.002	3	40
IQ 40% missing	As above			0.08 (0.039)	− 23	0.002	12	39
Correlation_(IQ: KS4)_ = 0.5				0.17 (0.034)	− 16	0.002	8	36
				0.27 (0.028)	− 12	0.002	9	37
IQ 40% missing	As above			0.08 (0.037)	− 17	0.002	27	32
Correlation_(IQ:KS4)_ = 0.7				0.18 (0.032)	− 11	0.002	20	28
				0.28 (0.027)	− 8	0.001	22	29
IQ 40% missing	As above			0.09 (0.032)	− 7	0.001	56	16
Correlation_(IQ:KS4)_ = 0.9				0.19 (0.029)	− 5	0.0009	44	14
				0.29 (0.025)	− 3	0.0007	50	14
IQ 60% missing	0.03 (0.049)	− 74	0.008	0.03 (0.050)	− 73	0.008	− 1	65
Correlation_(IQ:KS4)_ = 0.1	0.10 (0.043)	− 49	0.011	0.10 (0.043)	− 49	0.011	− 1	62
	0.19 (0.037)	− 36	0.013	0.19 (0.037)	− 35	0.013	− 1	63
IQ 60% missing	As above			0.03 (0.049)	− 68	0.007	0.6	64
Correlation_(IQ:KS4)_ = 0.3				0.11 (0.042)	− 46	0.01	1	61
				0.20 (0.037)	− 33	0.011	2	61
IQ 60% missing	As above			0.04 (0.047)	− 58	0.006	8	60
Correlation_(IQ:KS4)_ = 0.5				0.12 (0.041)	− 39	0.008	11	57
				0.22 (0.035)	− 28	0.008	12	57
IQ 60% missing	As above			0.06 (0.044)	− 42	0.004	28	52
Correlation_(IQ: KS4)_ = 0.7				0.14 (0.037)	− 28	0.004	31	48
				0.24 (0.032)	− 20	0.005	32	48
IQ 60% missing	As above			0.08 (0.036)	− 17	0.002	84	30
Correlation_(IQ:KS4)_ = 0.9				0.18 (0.032)	− 11	0.001	83	27
				0.28 (0.027)	− 8	0.001	86	28
IQ 80% missing	− 0.14 (0.068)	− 237	0.06	− 0.14 (0.069)	− 236	0.06	− 3	86
Correlation_(IQ: KS4)_ = 0.1	− 0.13 (0.062)	− 165	0.11	− 0.13 (0.062)	− 164	0.11	− 0.5	85
	− 0.05 (0.052)	− 116	0.12	− 0.05 (0.053)	− 115	0.12	− 1	85
IQ 80% missing	As above			− 0.12 (0.068)	− 223	0.05	− 0.3	85
Correlation_(IQ: KS4)_ = 0.3				− 0.11 (0.060)	− 155	0.1	6	84
				− 0.03 (0.051)	− 109	0.11	5	84
IQ 80% missing	As above			− 0.09 (0.065)	− 194	0.04	9	84
Correlation_(IQ: KS4)_ = 0.5				− 0.07 (0.056)	− 134	0.08	21	81
				0.02 (0.048)	− 94	0.08	19	82
IQ 80% missing	As above			− 0.04 (0.059)	− 143	0.02	36	79
Correlation_(IQ:KS4)_ = 0.7				0.002 (0.050)	− 99	0.04	56	76
				0.09 (0.043)	− 70	0.05	50	77
IQ 80% missing	As above			0.04 (0.044)	− 58	0.006	140	59
Correlation_(IQ:KS4)_ = 0.9				0.12 (0.038)	− 41	0.008	170	55
				0.21 (0.033)	− 29	0.009	156	57

As the correlation between the outcome and its proxy increased, the amount of information recovered through the imputations increased, thus reducing bias and increasing precision. Changing the correlation from 0.5 to 0.9 resulted in reductions of between 12 and 30% in the FMI (Table 5). Table 5 also shows that the FMI—and the resulting bias—was very similar with 40% missing data and a correlation of 0.7 between the original outcome and its linked proxy as in the scenario with 60% missing data and a correlation of 0.9; similarly, 80% missing data with a correlation of 0.9 resulted in a similar degree of bias to 60% missing data with a correlation of 0.5. With a very good proxy of the original outcome (i.e. with a correlation of 0.9), almost all the bias was eliminated, even with quite high proportions of missing data. Unsurprisingly, the bias was reduced when the strength of association between IQ and the probability of it being missing was reduced (Additional file 1: Table S1) and increased when the strength of this association was increased (Additional file 1: Table S2).

We introduced an interaction between the exposure and outcome with respect to the probability of being observed, such that the probability that IQ was observed was more strongly related to IQ itself among those who had not been breastfed compared to those who had. When this interaction was introduced the bias was exacerbated, particularly at higher levels of missing data. Nonetheless, the bias was reduced, and precision increased, through the use of MI incorporating the linkage data as auxiliary variables. These results are shown in the supplementary material (Additional file 1: Table S3).

Table 6 and Additional file 1: Table S4 show the results when missingness was introduced in the linked attainment score. When the correlation between the linked attainment score and IQ was 0.7 and the association between the linked variable and the probability of it being observed was in the opposite direction to the relationship between IQ and the probability of IQ being observed, the estimates were very similar to those obtained with no missing data for the linked attainment score. When the association was in the same direction as that for IQ, the estimates were slightly more biased than when there was no missingness in the linked data, except when there was 80% missing data; however, the differences were quite small and these estimates were still substantially less biased than those obtained from the complete records analysis (Table 6). When the correlation between the linked attainment score and IQ was 0.5, both sets of estimates were only slightly more biased than those obtained when there was no missingness in the linked data (Additional file 1: Table S4).

**Table 6:** Results when linked attainment score MNAR with 20% missing linked data (correlation between linked attainment score and IQ = 0.7); different values of difference in Pr(KS4 observed) for one SD increase in KS4 (diff Pr(KS4_obs_)) (factor 5 in scenarios)

Scenario [in each case: IQ MNAR (diff Pr(IQ obs) = 0.10), correlation_(IQ:KS4)_ = 0.7,	Complete records^a^			MI including linked attainment score (KS4)				
linked attainment = 20% missing]	Estimate (empirical SE)	% bias	MSE	Estimate (empirical SE)	% bias	MSE	Gain in precision (%)	FMI (%)
IQ 20% missing	0.08 (0.034)	− 17	0.001	0.09 (0.033)	− 7	0.001	8	17
Diff Pr(KS4_obs_) = − 0.10	0.17 (0.030)	− 14	0.002	0.19 (0.030)	− 7	0.001	6	14
	0.26 (0.025)	− 12	0.002	0.28 (0.026)	− 6	0.001	7	15
IQ 20% missing	As above			0.09 (0.033)	− 12	0.001	7	18
Diff Pr(KS4_obs_) = + 0.10				0.18 (0.030)	− 11	0.001	7	15
				0.27 (0.025)	− 9	0.001	9	15
IQ 40% missing,	0.07 (0.041)	− 29	0.003	0.09 (0.037)	− 15	0.002	16	34
Diff Pr(KS4_obs_) = − 0.10	0.16 (0.035)	−20	0.003	0.18 (0.034)	− 10	0.002	16	31
	0.26 (0.029)	− 15	0.003	0.28 (0.027)	− 8	0.001	17	32
IQ 40% missing	As above			0.08 (0.037)	− 20	0.002	22	35
Diff Pr(KS4_obs_) = + 0.10				0.17 (0.034)	− 15	0.002	15	32
				0.27 (0.028)	− 11	0.002	13	32
IQ 60% missing	0.03 (0.049)	− 74	0.008	0.06 (0.043)	− 43	0.004	32	55
Diff Pr(KS4_obs_) = − 0.10	0.10 (0.043)	− 49	0.01	0.14 (0.039)	− 30	0.005	24	52
	0.19 (0.037)	− 36	0.01	0.24 (0.032)	− 21	0.005	29	52
IQ 60% missing	As above			0.05 (0.042)	− 50	0.004	24	55
Diff Pr(KS4_obs_) = + 0.10				0.13 (0.037)	− 35	0.006	35	52
				0.23 (0.031)	− 25	0.006	33	52
IQ 80% missing	− 0.14 (0.068)	− 237	0.06	− 0.06 (0.060)	− 162	0.03	28	81
Diff Pr(KS4_obs_) = − 0.10	− 0.13 (0.062)	− 165	0.11	− 0.02 (0.054)	− 111	0.05	30	78
	− 0.05 (0.052)	− 116	0.12	0.07 (0.047)	− 78	0.06	25	80
IQ 80% missing	As above			− 0.06 (0.060)	− 156	0.03	37	80
Diff Pr(KS4_obs_) = + 0.10				− 0.01 (0.053)	− 108	0.05	36	77
				0.07 (0.045)	− 76	0.05	30	79

^a^The results for the complete records analysis presented here are the same as those presented in Table 5 but are included here for comparison

Finally, modelling the attainment score to be dependent on sex and mother’s education in addition to IQ in the simulated data had no discernible impact on the results (results not shown).

## Discussion

Our results illustrate how linkage to administrative data can enhance observational epidemiology. Linking to a proxy for a missing (continuous) study outcome which is MNAR, and including this as an auxiliary variable in multiple imputation models can reduce bias and increase precision when the correlation between the study outcome and the linked proxy is relatively high (r ≥ 0.5), even with very high levels of missing data (80%). Inclusion of the proxy variable means that the outcome is a better approximation to MAR, particularly when there is a high correlation between the proxy and the original outcome. In our analysis of the ALSPAC data we showed that including more than one linked variable can result in small additional gains, both in terms of precision and bias. The inclusion of more than one proxy variable is likely to result in larger gains if these variables are relatively independent predictors of the original study outcome.

In the ALSPAC dataset there were 4152 complete records out of a sample of 13,975 subjects, so 70% had missing data. The attainment variables explained 44% of the variability in IQ among individuals with all of these measures available. This information, together with the results from our simulations, suggest that we are still likely to have under-estimated the impact of duration of breastfeeding on IQ in our analysis.

Our results also highlight the relevance of the FMI—as opposed to simply the response rate—as a guide to the level of missing information and resulting uncertainty in the (analysis of the) imputed data. This has been highlighted previously [9]. Wagner [10] demonstrated how the FMI could be useful both in terms of monitoring and designing surveys. Others have discussed its use as a tool to select auxiliary variables for inclusion in imputation models [11]. In our simulated datasets, having a linked proxy (attainment score) that was strongly correlated with the original outcome resulted in an FMI (and bias) of a similar magnitude to that found in a dataset with a much lower percentage of missing data but with a poorer proxy variable. Linking to external datasets is not always straightforward and can be costly; however, if the linked data are likely to contain auxiliary variables that are highly correlated with study outcomes, the benefits in terms of reductions in bias and FMI and increased precision may outweigh the costs. This is likely to particularly the case when there is a high proportion of missing data in the outcome of interest.

Any source of linked data is unlikely to have complete population coverage and, in circumstances where linkage to administrative data requires consent, this may not be obtained for all participants. In our example, we linked to the National Pupil Database, which covers only schools in England that follow the National Curriculum. This could impact on the potential benefits of obtaining linked data. However, we simulated a relatively modest amount of missingness in the linked data and showed that, even when individuals with higher probabilities of having missing outcome data were also more likely to have missing linked data, this had little impact on the results. Thus, even in situations with incomplete coverage of the linked datasets, use of a linked proxy is likely to result in gains in efficiency and reductions in bias.

**Fig. 1:**
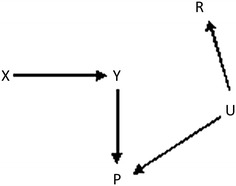
DAG illustrating a scenario in which inclusion of a proxy may increase bias

Our study has some limitations. We covered a range of plausible scenarios in our simulations but did not consider every possible situation. For example, in our simulations we simulated missingness in IQ to be linearly related to the value of IQ. Further, in the ALSPAC data there were non-linear relationships between IQ and the linked attainment scores; we did not incorporate this non-linearity in the simulation models because we did not want to make the simulations too specific to this particular example. Further, in our simulations we assumed that missingness in IQ was fully explained by the covariates and itself. If there were one or more unmeasured factors predictive of missingness then the relative reductions in bias would be lower. If the proxy were strongly associated with these unmeasured factor(s) then use of the proxy could either increase or reduce bias, depending on the size and directions of all the associations. We agree with the recommendation by Thoemmes and Rose [12] that careful thought is given to the causal structure between the variables included in the analysis model, the potential proxy variables, and the missingness mechanism in order to identify whether inclusion of a particular proxy is likely to increase or decrease bias. For example, the directed acyclic graph (DAG) shown below illustrates a situation in which the study outcome is not actually MNAR but missingness depends on the proxy (this could be directly or via an unmeasured factor denoted by U). In this situation, inclusion of the proxy in the multiple imputation model would induce an association between the study outcome and missingness (R) and thus increase bias. Clearly, the extent of bias will depend on the exact scenario. For a given applied situation, tailored simulations could be used to assess the likely extent of bias due to MNAR (Fig. 1).

When missing data occurs only in the outcome variable then, in the absence of auxiliary variables, a complete records analysis and multiple imputation will produce essentially the same results [13, 14]. As such, it is generally thought that, unless there are auxiliary variables that are at least moderately correlated with the outcome of interest, then there is little to be gained by using multiple imputation [15]. These auxiliary variables could be, as in our example, proxy measures of the outcome variable obtained via linkage to external datasets or could be earlier (or later) measurements of the same, or a similar, outcome. Other studies have examined the impact of the inclusion of auxiliary variables in MI models. Collins et al. [16] found—like us—that in the scenarios they investigated, the addition of auxiliary variables that were predictors of missingness in the outcome in MI models increased efficiency and reduced bias, even when the correlation between the auxiliary variable and the original outcome variable was relatively low (0.4). However, in their study the simulations were designed such that the data were MNAR when the auxiliary variables were omitted but MAR when they were included. As such, the MI models including the auxiliary variables would be unbiased by design. More recently, Mustillo and Kwon [17] found that the inclusion of auxiliary variables increased efficiency by quite small amounts but did not always reduce bias when data were MNAR. Further, they found that the bias resulting from the data being MNAR was small. This could perhaps be explained by the fact that the correlation between their exposure and their outcome in their simulated data was quite high (0.6); further, they only considered 10, 20 and 30% missing data. In the dataset in which they simulated missingness, it is not stated how strongly other covariates were related to the exposure variable which they simulated as being MNAR but, again, they only considered 10–30% missing data. Two other studies [18, 19] used data available from medical records as auxiliary variables in MI models when the outcome variable was MNAR; using these data plus results from simulations, both studies found that the inclusion of these auxiliary variables reduced bias but did not completely eliminate it. However, in both of these studies they were looking at bias in the marginal distribution of the outcome variable itself rather than in adjusted estimates of the association between an exposure and the missing outcome.

## Conclusions

In a study where an outcome variable is MNAR, proxies for this outcome obtained from linked administrative or other external datasets should be incorporated as auxiliary variables in multiple imputation models if they have reasonably high correlations—either individually or jointly—with the study outcome. We strongly recommend this strategy, as their inclusion will reduce bias and increase efficiency under a wide range of conditions, even with high levels of missing data, and even when the linked data are themselves MNAR. That said, it is important to consider the causal relationships between the study outcome, its proxies, and missingness, as there may be situations in which inclusion of proxies will increase bias. Where such proxies are not available, simulations designed for the particular situation being studied should be used as sensitivity analyses to examine the potential degree of bias.

## Additional file

**Additional file 1.**

## Abbreviations

MNAR: missing not at random
MAR: missing at random
MI: multiple imputation
ALSPAC: Avon Longitudinal Study of Parents and Children
NPD: National Pupil Database
WASI: Wechsler Abbreviated Scale of Intelligence
KS4, KS3, KS2: Key Stage 4, 3, 2
GCSE: General Certificate of Secondary Education
FMI: fraction of missing information
