# Supplementary material for: Multiple imputation using linked proxy outcome data resulted in important bias reduction and efficiency gains: a simulation study
Source: Emerg Themes Epidemiol. 2017 Dec 19;14:14. doi: 10.1186/s12982-017-0068-0 (PMC5735815; doi:10.1186/s12982-017-0068-0)
Supplement: Supplementary file 1 — Additional file 1. [file 12982_2017_68_MOESM1_ESM.docx]

Supplementary Tables

Supplementary Table 1: Results for IQ MNAR: difference in Pr(IQ observed) = 0.05 for 1 SD increase in IQ (factor 3 in scenarios)

|  | Complete records | | | MI including linked attainment score (KS4) | | | | |
| --- | --- | --- | --- | --- | --- | --- | --- | --- |
| Scenario  (factors 1 and 2) | Estimate (empirical SE) | % bias | MSE | Estimate (empirical SE) | % bias | MSE | Gain in precision | FMI |
| IQ 20% missing  Correlation_(IQ:KS4)_ = 0.1 | 0.09 (0.034)  0.19 (0.031)  0.29 (0.025) | -6%  -5%  -4% | 0.001  0.001  0.0008 | 0.09 (0.034)  0.19 (0.031)  0.29 (0.025) | -5%  -4%  -4% | 0.001  0.001  0.0008 | 0.1%  0.1%  0.2% | 24%  21%  22% |
| IQ 20% missing  Correlation_(IQ:KS4)_ = 0.3 | As above | | | 0.09 (0.034)  0.19 (0.031)  0.29 (0.025) | -5%  -4%  -4% | 0.001  0.001  0.0008 | 1.8%  1.3%  1.2% | 23%  20%  20% |
| IQ 20% missing  Correlation_(IQ:KS4)_ = 0.5 | As above | | | 0.10 (0.033)  0.19 (0.030)  0.29 (0.025) | -4%  -4%  -3% | 0.001  0.001  0.0007 | 6%  4%  3% | 20%  17%  18% |
| IQ 20% missing  Correlation_(IQ:KS4)_ = 0.7 | As above | | | 0.10 (0.032)  0.20 (0.029)  0.29 (0.024) | -3%  -3%  -2% | 0.001  0.0009  0.0006 | 14%  8%  8% | 15%  13%  13% |
| IQ 20% missing  Correlation_(IQ:KS4)_ = 0.9 | As above | | | 0.10 (0.031)  0.20 (0.029)  0.30 (0.024) | -1%  -1%  -1% | 0.0009  0.0008  0.0006 | 27%  16%  15% | 7%  6%  6% |
| IQ 40% missing  Correlation_(IQ: KS4)_ = 0.1 | 0.09 (0.039)  0.18 (0.035)  0.28 (0.030) | -11%  -9%  -6% | 0.002  0.002  0.001 | 0.09 (0.039)  0.18 (0.036)  0.28 (0.030) | -11%  -9%  -6% | 0.002  0.002  0.001 | -0.4%  -0.5%  0.1% | 44%  41%  42% |
| IQ 40% missing  Correlation_(IQ: KS4)_ = 0.3 | As above | | | 0.09 (0.039)  0.18 (0.035)  0.28 (0.029) | -10%  -8%  -5% | 0.002  0.002  0.001 | 3.3%  1.5%  3.2% | 43%  40%  40% |
| IQ 40% missing  Correlation_(IQ: KS4)_ = 0.5 | As above | | | 0.09 (0.037)  0.19 (0.034)  0.29 (0.028) | -9%  -7%  -5% | 0.001  0.001  0.001 | 11%  8%  10% | 38%  35%  36% |
| IQ 40% missing  Correlation_(IQ:KS4)_ = 0.7 | As above | | | 0.09 (0.035)  0.19 (0.032)  0.29 (0.027) | -6%  -6%  -3% | 0.001  0.001  0.0008 | 26%  20%  22% | 31%  28%  29% |
| IQ 40% missing  Correlation_(IQ:KS4)_ = 0.9 | As above | | | 0.10 (0.032)  0.19 (0.029)  0.29 (0.024) | -3%  -3%  -1% | 0.001  0.0009  0.0006 | 54%  46%  48% | 15%  13%  14% |
| IQ 60% missing  Correlation_(IQ:KS4)_ = 0.1 | 0.07 (0.049)  0.15 (0.044)  0.25 (0.038) | -32%  -23%  -16% | 0.003  0.004  0.004 | 0.07 (0.049)  0.16 (0.042)  0.25 (0.038) | -32%  -22%  -15% | 0.003  0.004  0.004 | -1.2%  -0.4%  0.3% | 65%  62%  63% |
| IQ 60% missing  Correlation_(IQ:KS4)_ = 0.3 | As above | | | 0.07 (0.048)  0.16 (0.041)  0.26 (0.037) | -29%  -21%  -14% | 0.003  0.003  0.003 | 3.0%  3.1%  5.1% | 63%  61%  61% |
| IQ 60% missing  Correlation_(IQ:KS4)_ = 0.5 | As above | | | 0.08 (0.046)  0.16 (0.041)  0.25 (0.036) | -25%  -18%  -12% | 0.003  0.003  0.003 | 14%  12%  16% | 59%  56%  57% |
| IQ 60% missing  Correlation_(IQ: KS4)_ = 0.7 | As above | | | 0.08 (0.042)  0.17 (0.039)  0.27 (0.032) | -17%  -13%  -8% | 0.002  0.002  0.002 | 35%  30%  40% | 50%  47%  48% |
| IQ 60% missing  Correlation_(IQ:KS4)_ = 0.9 | As above | | | 0.09 (0.035)  0.19 (0.031)  0.29 (0.027) | -6%  -7%  -3% | 0.001  0.001  0.0008 | 93%  79%  102% | 29%  26%  27% |
| IQ 80% missing  Correlation_(IQ: KS4)_ = 0.1 | -0.09 (0.075)  -0.03 (0.063)  0.06 (0.055) | -187%  -117%  -79% | 0.04  0.06  0.06 | -0.08 (0.074)  -0.03 (0.063)  0.07 (0.055) | -185%  -116%  -78% | 0.04  0.06  0.06 | 0.2%  0.3%  -0.2% | 85%  84%  85% |
| IQ 80% missing  Correlation_(IQ: KS4)_ = 0.3 | As above | | | -0.07 (0.072)  -0.02 (0.061)  0.08 (0.053) | -173%  -113%  -73% | 0.04  0.05  0.05 | 6.0%  5.7%  4.4% | 84%  83%  84% |
| IQ 80% missing  Correlation_(IQ: KS4)_ = 0.5 | As above | | | -0.05 (0.068)  0.01 (0.058)  0.11 (0.051) | -148%  -93%  -63% | 0.03  0.04  0.04 | 20%  18%  16% | 82%  80%  82% |
| IQ 80% missing  Correlation_(IQ:KS4)_ = 0.7 | As above | | | -0.007 (0.061)  0.07 (0.052)  0.16 (0.045) | -107%  -67%  -45% | 0.02  0.02  0.02 | 54%  48%  48% | 77%  75%  76% |
| IQ 80% missing  Correlation_(IQ:KS4)_ = 0.9 | As above | | | 0.06 (0.045)  0.15 (0.039)  0.25 (0.034) | -43%  -27%  -18% | 0.003  0.004  0.004 | 172%  157%  164% | 57%  54%  56% |

Supplementary Table 2: Results for IQ MNAR: difference in Pr(IQ observed) = 0.20 for 1 SD increase in IQ (factor 3 in scenarios)

|  | Complete records | | | MI including linked attainment score | | | | |
| --- | --- | --- | --- | --- | --- | --- | --- | --- |
| Scenario  (factors 1 and 2) | Estimate (empirical SE) | % bias | MSE | Estimate (empirical SE) | % bias | MSE | Gain in precision | FMI |
| IQ 20% missing  Correlation_(IQ:KS4)_ = 0.1 | 0.05 (0.032)  0.13 (0.030)  0.21 (0.024) | -47%  -38%  -30% | 0.003  0.007  0.009 | 0.05 (0.032)  0.13 (0.030)  0.21 (0.024) | -47%  -37%  -30% | 0.003  0.006  0.009 | 0.1%  0.7%  0.5% | 24%  21%  21% |
| IQ 20% missing  Correlation_(IQ:KS4)_ = 0.3 | As above | | | 0.06 (0.032)  0.13 (0.030)  0.22 (0.024) | -44%  -35%  -28% | 0.003  0.006  0.008 | 0.9%  2.8%  2.3% | 23%  20%  20% |
| IQ 20% missing  Correlation_(IQ:KS4)_ = 0.5 | As above | | | 0.06 (0.031)  0.14 (0.029)  0.23 (0.024) | -38%  -30%  -24% | 0.002  0.004  0.006 | 4%  6%  5% | 21%  17%  17% |
| IQ 20% missing  Correlation_(IQ:KS4)_ = 0.7 | As above | | | 0.07 (0.031)  0.16 (0.029)  0.25 (0.023) | -28%  -22%  -17% | 0.002  0.002  0.003 | 9%  12%  9% | 16%  13%  13% |
| IQ 20% missing  Correlation_(IQ:KS4)_ = 0.9 | As above | | | 0.09 (0.029)  0.18 (0.028)  0.28 (0.023) | -12%  -9%  -7% | 0.001  0.001  0.001 | 18%  18%  13% | 7%  6%  6% |
| IQ 40% missing  Correlation_(IQ: KS4)_ = 0.1 | 0.03 (0.039)  0.10 (0.033)  0.19 (0.029) | -67%  -49%  -38% | 0.006  0.011  0.014 | 0.03 (0.039)  0.10 (0.033)  0.19 (0.029) | -66%  -48%  -38% | 0.006  0.010  0.013 | -0.2%  0.7%  0.7% | 47%  42%  42% |
| IQ 40% missing  Correlation_(IQ: KS4)_ = 0.3 | As above | | | 0.04 (0.038)  0.11 (0.033)  0.19 (0.028) | -62%  -45%  -35% | 0.005  0.009  0.012 | 2.0%  3.3%  2.1% | 45%  41%  41% |
| IQ 40% missing  Correlation_(IQ: KS4)_ = 0.5 | As above | | | 0.05 (0.037)  0.12 (0.032)  0.21 (0.027) | -53%  -39%  -30% | 0.004  0.007  0.009 | 7%  8%  8% | 41%  37%  37% |
| IQ 40% missing  Correlation_(IQ:KS4)_ = 0.7 | As above | | | 0.06 (0.036)  0.14 (0.031)  0.23 (0.026) | -39%  -29%  -22% | 0.003  0.004  0.005 | 17%  17%  19% | 33%  30%  30% |
| IQ 40% missing  Correlation_(IQ:KS4)_ = 0.9 | As above | | | 0.08 (0.033)  0.18 (0.029)  0.27 (0.024) | -16%  -12%  -9% | 0.001  0.001  0.001 | 38%  35%  39% | 16%  14%  14% |
| IQ 60% missing  Correlation_(IQ: KS4)_ = 0.1 | -0.02 (0.047)  0.02 (0.041)  0.10 (0.035) | -125%  -92%  -67% | 0.018  0.035  0.042 | -0.02 (0.047)  0.02 (0.041)  0.10 (0.035) | -124%  -90%  -66% | 0.017  0.034  0.041 | 0.1%  0.4%  0.4% | 67%  64%  64% |
| IQ 60% missing  Correlation_(IQ: KS4)_ = 0.3 | As above | | | -0.02 (0.046)  0.03 (0.040)  0.11 (0.034) | -116%  -85%  -63% | 0.016  0.031  0.036 | 4.2%  4.5%  4.3% | 66%  63%  63% |
| IQ 60% missing  Correlation_(IQ:KS4)_ = 0.5 | As above | | | -0.0006 (0.044)  0.05 (0.039)  0.14 (0.033) | -101%  -74%  -54% | 0.012  0.022  0.028 | 14%  13%  13% | 63%  59%  59% |
| IQ 60% missing  Correlation_(IQ: KS4)_ = 0.7 | As above | | | 0.03 (0.040)  0.09 (0.036)  0.18 (0.030) | -74%  -55%  -40% | 0.007  0.013  0.016 | 34%  32%  30% | 54%  50%  51% |
| IQ 60% missing  Correlation_(IQ:KS4)_ = 0.9 | As above | | | 0.07 (0.034)  0.15 (0.031)  0.25 (0.026) | -31%  -24%  -17% | 0.002  0.003  0.003 | 85%  78%  73% | 32%  28%  29% |
| IQ 80% missing  Correlation_(IQ: KS4)_ = 0.1 | -0.12 (0.064)  -0.13 (0.054)  -0.08 (0.045) | -219%  -166%  -125% | 0.05  0.11  0.14 | -0.12 (0.064)  -0.13 (0.054)  -0.07 (0.046) | -219%  -165%  -125% | 0.05  0.11  0.14 | -0.3%  -0.9%  -0.3% | 86%  85%  85% |
| IQ 80% missing  Correlation_(IQ: KS4)_ = 0.3 | As above | | | -0.11 (0.063)  -0.11 (0.053)  -0.06 (0.045) | -208%  -157%  -119% | 0.05  0.10  0.13 | 3.2%  2.6%  2.5% | 86%  84%  84% |
| IQ 80% missing  Correlation_(IQ: KS4)_ = 0.5 | As above | | | -0.08 (0.060)  -0.08 (0.051)  -0.02 (0.043) | -184%  -139%  -105% | 0.04  0.08  0.10 | 13%  11%  10% | 84%  82%  83% |
| IQ 80% missing  Correlation_(IQ:KS4)_ = 0.7 | As above | | | -0.04 (0.055)  -0.01 (0.047)  0.06 (0.040) | -142%  -107%  -81% | 0.02  0.05  0.06 | 36%  31%  29% | 80%  77%  78% |
| IQ 80% missing  Correlation_(IQ:KS4)_ = 0.9 | As above | | | 0.04 (0.043)  0.11 (0.038)  0.19 (0.033) | -64%  -48%  -37% | 0.006  0.01  0.01 | 120%  96%  94% | 61%  57%  58% |

Supplementary Table 3: Results for IQ MNAR with an interaction between breastfeeding and IQ with respect to the probability of IQ being observed: difference in Pr(IQ observed) = 0.10 for 1 SD increase in IQ when exposure=0 (no breastfeeding); change in difference in Pr(IQ observed) for each 1 SD increase in IQ = -0.025 for each increase in breastfeeding category (factor 4 in scenarios)

|  | Complete records | | | MI including linked attainment score (KS4) | | | | |
| --- | --- | --- | --- | --- | --- | --- | --- | --- |
| Scenario  (factors 1 and 2) | Estimate (empirical SE) | % bias | MSE | Estimate (empirical SE) | % bias | MSE | Gain in precision | FMI |
| IQ 20% missing  Correlation_(IQ:KS4)_ = 0.1 | 0.06 (0.034)  0.13 (0.031)  0.21 (0.026) | -42%  -36%  -31% | 0.003  0.006  0.009 | 0.06 (0.034)  0.13 (0.031)  0.21 (0.026) | -41%  -36%  -30% | 0.003  0.006  0.009 | 0.6%  0.3%  0.5% | 24%  21%  21% |
| IQ 20% missing  Correlation_(IQ:KS4)_ = 0.3 | As above | | | 0.06 (0.034)  0.13 (0.030)  0.22 (0.026) | -39%  -33%  -28% | 0.003  0.005  0.008 | 3%  2%  2% | 23%  20%  20% |
| IQ 20% missing  Correlation_(IQ:KS4)_ = 0.5 | As above | | | 0.07 (0.033)  0.14 (0.030)  0.23 (0.025) | -33%  -28%  -24% | 0.002  0.004  0.006 | 8%  6%  5% | 24%  21%  21% |
| IQ 20% missing  Correlation_(IQ:KS4)_ = 0.7 | As above | | | 0.08 (0.032)  0.16 (0.029)  0.25 (0.025) | -24%  -20%  -17% | 0.002  0.002  0.003 | 15%  12%  11% | 15%  13%  13% |
| IQ 20% missing  Correlation_(IQ:KS4)_ = 0.9 | As above | | | 0.09 (0.030)  0.18 (0.028)  0.28 (0.024) | -9%  -8%  -7% | 0.001  0.001  0.001 | 27%  20%  19% | 7%  5%  6% |
| IQ 40% missing  Correlation_(IQ: KS4)_ = 0.1 | 0.03 (0.040)  0.10 (0.036)  0.17 (0.030) | -66%  -51%  -44% | 0.006  0.012  0.019 | 0.03 (0.040)  0.10 (0.036)  0.17 (0.030) | -65%  -51%  -44% | 0.006  0.011  0.018 | -0.5%  0.3%  -0.5% | 44%  41%  41% |
| IQ 40% missing  Correlation_(IQ: KS4)_ = 0.3 | As above | | | 0.04 (0.039)  0.11 (0.035)  0.18 (0.030) | -61%  -47%  -41% | 0.005  0.010  0.016 | 3%  4%  2% | 42%  40%  40% |
| IQ 40% missing  Correlation_(IQ: KS4)_ = 0.5 | As above | | | 0.05 (0.038)  0.12 (0.034)  0.20 (0.029) | -52%  -40%  -35% | 0.004  0.008  0.012 | 10%  10%  9% | 38%  36%  36% |
| IQ 40% missing  Correlation_(IQ: KS4)_ = 0.7 | As above | | | 0.06 (0.036)  0.14 (0.032)  0.23 (0.027) | -37%  -29%  -25% | 0.003  0.004  0.006 | 23%  24%  22% | 31%  28%  29% |
| IQ 40% missing  Correlation_(IQ:KS4)_ = 0.9 | As above | | | 0.08 (0.032)  0.18 (0.029)  0.28 (0.025) | -15%  -11%  -10% | 0.001  0.001  0.001 | 49%  50%  51% | 15%  13%  14% |
| IQ 60% missing  Correlation_(IQ:KS4)_ = 0.1 | -0.03 (0.048)  0.003 (0.043)  0.06 (0.037) | -132%  -99%  -79% | 0.020  0.041  0.058 | -0.03 (0.048)  0.005 (0.043)  0.06 (0.037) | -131%  -98%  -79% | 0.019  0.040  0.057 | -0.5%  -0.7%  0.1% | 63%  63%  63% |
| IQ 60% missing  Correlation_(IQ:KS4)_ = 0.3 | As above | | | -0.02 (0.048)  0.02 (0.043)  0.08 (0.037) | -123%  -91%  -73% | 0.017  0.035  0.050 | 2%  2%  4% | 63%  61%  61% |
| IQ 60% missing  Correlation_(IQ:KS4)_ = 0.5 | As above | | | -0.005 (0.046)  0.04 (0.041)  0.11 (0.035) | -105%  -78%  -63% | 0.013  0.026  0.036 | 11%  11%  15% | 59%  57%  57% |
| IQ 60% missing  Correlation_(IQ:KS4)_ = 0.7 | As above | | | 0.02 (0.042)  0.09 (0.038)  0.17 (0.032) | -76%  -56%  -45% | 0.008  0.014  0.019 | 30%  30%  38% | 51%  48%  49% |
| IQ 60% missing  Correlation_(IQ: KS4)_ = 0.9 | As above | | | 0.07 (0.035)  0.16 (0.032)  0.26 (0.026) | -31%  -22%  -18% | 0.002  0.003  0.004 | 85%  79%  99% | 29%  27%  28% |
| IQ 80% missing  Correlation_(IQ: KS4)_ = 0.1 | -0.23 (0.070)  -0.28 (0.062)  -0.25 (0.054) | -330%  -242%  -184% | 0.11  0.24  0.31 | -0.23 (0.070)  -0.28 (0.062)  -0.25 (0.054) | -328%  -240%  -183% | 0.11  0.23  0.30 | 1%  -0.5%  0.3% | 85%  84%  84% |
| IQ 80% missing  Correlation_(IQ: KS4)_ = 0.3 | As above | | | -0.21 (0.068)  -0.25 (0.061)  -0.21 (0.052) | -307%  -225%  -171% | 0.10  0.21  0.27 | 6%  4%  5% | 84%  83%  83% |
| IQ 80% missing  Correlation_(IQ: KS4)_ = 0.5 | As above | | | -0.16 (0.064)  -0.19 (0.058)  -0.14 (0.050) | -263%  -193%  -147% | 0.07  0.15  0.20 | 18%  14%  16% | 82%  80%  81% |
| IQ 80% missing  Correlation_(IQ: KS4)_ = 0.7 | As above | | | -0.09 (0.058)  -0.08 (0.052)  -0.02 (0.045) | -190%  -140%  -106% | 0.04  0.08  0.10 | 47%  41%  43% | 77%  74%  76% |
| IQ 80% missing  Correlation_(IQ:KS4)_ = 0.9 | As above | | | 0.02 (0.044)  0.09 (0.040)  0.17 (0.034) | -78%  -57%  -43% | 0.008  0.01  0.02 | 147%  135%  147% | 57%  53%  56% |

Supplementary Table 4: Results when linked attainment score MNAR with 20% missing linked data (and correlation between linked attainment score and IQ = 0.5); different values of difference in Pr(KS4 observed) for one SD increase in KS4 (diff Pr(KS4_obs_)) (factor 5 in scenarios)

| Scenario [in each case: IQ MNAR (diff Pr(IQ obs) = 0.10),  correlation_(IQ:KS4)_ = 0.5,  linked attainment = 20% missing] | Complete records^1^ | | | MI including linked attainment score (KS4) | | | | |
| --- | --- | --- | --- | --- | --- | --- | --- | --- |
|  | Estimate (empirical SE) | % bias | MSE | Estimate (empirical SE) | % bias | MSE | Gain in precision | FMI |
| IQ 20% missing  Diff Pr(KS4_obs_) = -0.10 | 0.08 (0.034)  0.17 (0.030)  0.26 (0.025) | -17%  -14%  -12% | 0.001  0.002  0.002 | 0.09 (0.033)  0.18 (0.029)  0.27 (0.025) | -15%  -12%  -11% | 0.001  0.002  0.002 | 4%  2%  2% | 21%  18%  18% |
| IQ 20% missing  Diff Pr(KS4_obs_) = +0.10 | As above | | | 0.09 (0.034)  0.17 (0.030)  0.27 (0.025) | -14%  -13%  -11% | 0.001  0.002  0.002 | 4%  2%  3% | 21%  18%  18% |
| IQ 40% missing,  Diff Pr(KS4_obs_) = -0.10 | 0.07 (0.041)  0.16 (0.035)  0.26 (0.029) | -29%  -20%  -15% | 0.003  0.003  0.003 | 0.08 (0.038)  0.17 (0.034)  0.27 (0.028) | -23%  -17%  -11% | 0.002  0.002  0.002 | 10%  7%  8% | 41%  37%  38% |
| IQ 40% missing  Diff Pr(KS4_obs_) = +0.10 | As above | | | 0.08 (0.038)  0.17 (0.035)  0.26 (0.030) | -24%  -16%  -13% | 0.002  0.002  0.002 | 8%  9%  6% | 41%  37%  38% |
| IQ 60% missing  Diff Pr(KS4_obs_) = -0.10 | 0.03 (0.049)  0.10 (0.043)  0.19 (0.037) | -74%  -49%  -36% | 0.008  0.01  0.01 | 0.04 (0.045)  0.12 (0.041)  0.21 (0.035) | -60%  -42%  -29% | 0.006  0.009  0.009 | 12%  11%  10% | 61%  58%  59% |
| IQ 60% missing  Diff Pr(KS4_obs_) = +0.10 | As above | | | 0.04 (0.046)  0.12 (0.040)  0.21 (0.034) | -63%  -42%  -29% | 0.006  0.007  0.006 | 10%  11%  15% | 61%  58%  59% |
| IQ 80% missing  Diff Pr(KS4_obs_) = -0.10 | -0.14 (0.068)  -0.13 (0.062)  -0.05 (0.052) | -237%  -165%  -116% | 0.06  0.11  0.12 | -0.10 (0.065)  -0.08 (0.056)  0.003 (0.051) | -204%  -141%  -99% | 0.05  0.08  0.09 | 15%  13%  10% | 84%  82%  83% |
| IQ 80% missing  Diff Pr(KS4_obs_) = +0.10 | As above | | | -0.10 (0.065)  -0.08 (0.059)  0.005 (0.049) | -201%  -140%  -98% | 0.04  0.08  0.09 | 11%  16%  11% | 82%  83%  80% |

1. The results for the complete records analysis presented here are the same as those presented in Table 5 but are included here for comparison.
